# Histological examination of carotid artery tissue in cases of ligature strangulation and hanging

**DOI:** 10.1080/20961790.2022.2034715

**Published:** 2022-03-14

**Authors:** Julia Ulbricht, Burkhard Madea, Elke Doberentz

**Affiliations:** Institute of Legal Medicine, University Hospital Bonn, Bonn, Germany

**Keywords:** Forensic sciences, carotid bifurcation, carotid body, carotid sinus, haemorrhage, hanging, ligature strangulation, aquaporin-3 (AQP3), heat-shock protein (Hsp)

## Abstract

Violence against the neck can result in a range of macromorphological and micromorphological findings. However, the forensic relevance of the carotid sinus in cases of violence against the neck remains controversial. In this follow-up study of 22 cases of suicidal and accidental strangulations, carotid bifurcations were examined histologically for morphological changes implying direct trauma, including haemorrhage and immunohistochemical expression of heat-shock proteins 27, 60, and 70 and aquaporin-3. These cases were compared with a control group (82 cases) without neck compression or head trauma and with variable causes of death. No relevant histopathological findings implying direct trauma of the carotid bifurcation were found. No cases showed positive aquaporin-3 staining and only five cases showed positive heat-shock protein-27 staining, all of which were hangings. Without massive trauma of the carotid bifurcation, histological alterations cannot be expected. Without signs of rapid death, findings of acute circulatory failure, macromorphological and micromorphological findings of neck compression, and reliable markers indicating relevant impact on the carotid bifurcation the diagnosis of a lethal reflex cannot be verified.

Among 22 cases of strangulation causing death, there were 16 cases of hanging and 6 cases of ligature strangulation

Few cases showed small haemorrhages located predominantly in the surrounding fat and connective tissues; however, the haemorrhages did not have any effects on the tissues

Neck compression had minimal effects on heat shock protein 27 expression in carotid artery tissue

Aquaporin-3 staining suggested it is not a useful marker for relevant neck pressure, or that there had not been any relevant neck impact

Our findings suggested no direct evidence for reflex cardiac death resulting from a brief force against the neck

## Introduction

The carotid bifurcation is involved in the regulation of blood circulation because of the presence of the carotid body and sinus. The carotid body is located within the bifurcation, between the internal and external carotid arteries, while the carotid sinus is located inside the vessel wall of the internal artery. Stimulation of either structure (e.g. by hypoxia or vasoconstriction) can induce changes in heart rate and blood pressure *via* chemoreceptor and pressoreceptor activity [1, 2]. The carotid sinus can also be stimulated by external pressure, leading to vessel wall deformation. The faster and more extensive the stimulation, the more extensive the inhibition of afferent sympathetic fibres and the stimulation of parasympathetic fibres, resulting in hypotension and bradycardia [3]. This effect is used clinically (termed the carotid sinus massage) to interrupt tachyarrhythmias, which rarely result in asystole [4, 5]. This reflex predominantly causes cardioinhibitory and vasodepressive effects [6]. Therefore, stimulation, particularly of the carotid sinus, can induce bradycardia up to asystole with circulatory failure [7–9]. In young and healthy subjects, unilateral pressure typically only causes a minor decrease in heart rate and blood pressure [10], although induced ventricular fibrillation has been reported in a few cases [11–13]. Most forensic pathologists believe that this cardioinhibitory reflex can, at least in theory, cause death [14].

In 1927, the German physiologist Heinrich Ewald Hering theorised that the carotid sinus may be affected in cases of violence against the neck. For example, in cases of hanging, a carotid sinus reflex with decrease in heart rate and blood pressure and rapid loss of consciousness may occur. However, in people with arteriosclerosis, light pressure to the neck is sufficient to induce unconsciousness. Nevertheless, his studies were predominantly performed on animals [15]. Therefore, the mechanisms by which the cardioinhibitory reflex occurs and whether evidence of a lethal reflex can be verified during human autopsy remains unknown. Furthermore, whether a minor neck trauma such as a short grip to the neck can cause sudden loss of consciousness and even death because of a carotid sinus reflex is unclear.

Herein, we performed a follow-up study of cases of death due to ligature strangulation and hanging. The study aimed to assess histological macroscopic findings (particularly haemorrhage in tissues surrounding the carotid bifurcations, the sinus, and the carotid body as potential direct indicators of tissue trauma and a cardiac reflex and expression of heat-shock proteins (Hsp) 27, 60, and 70 and aquaporin-3 (AQP-3) [16]), as well as the patients’ history.

## Materials and methods

The study group consisted of 22 cases of ligature strangulation and hanging, in which autopsy was performed at our Institute of Legal Medicine. All available information was obtained from autopsy protocols or documentations by investigation authorities. The study group comprised 7 female and 15 male individuals, with a mean age of 54.04 years (range, 10–91 years). The control group comprised 82 cases with natural and nonnatural causes of death, including myocardial infarction or head shot wounds, with different agonal periods but without evidence of neck trauma [17]. The control group comprised 55 female and 27 male individuals, with a mean age of 55.2 years (range, 17–85 years).

**Figure 1.**
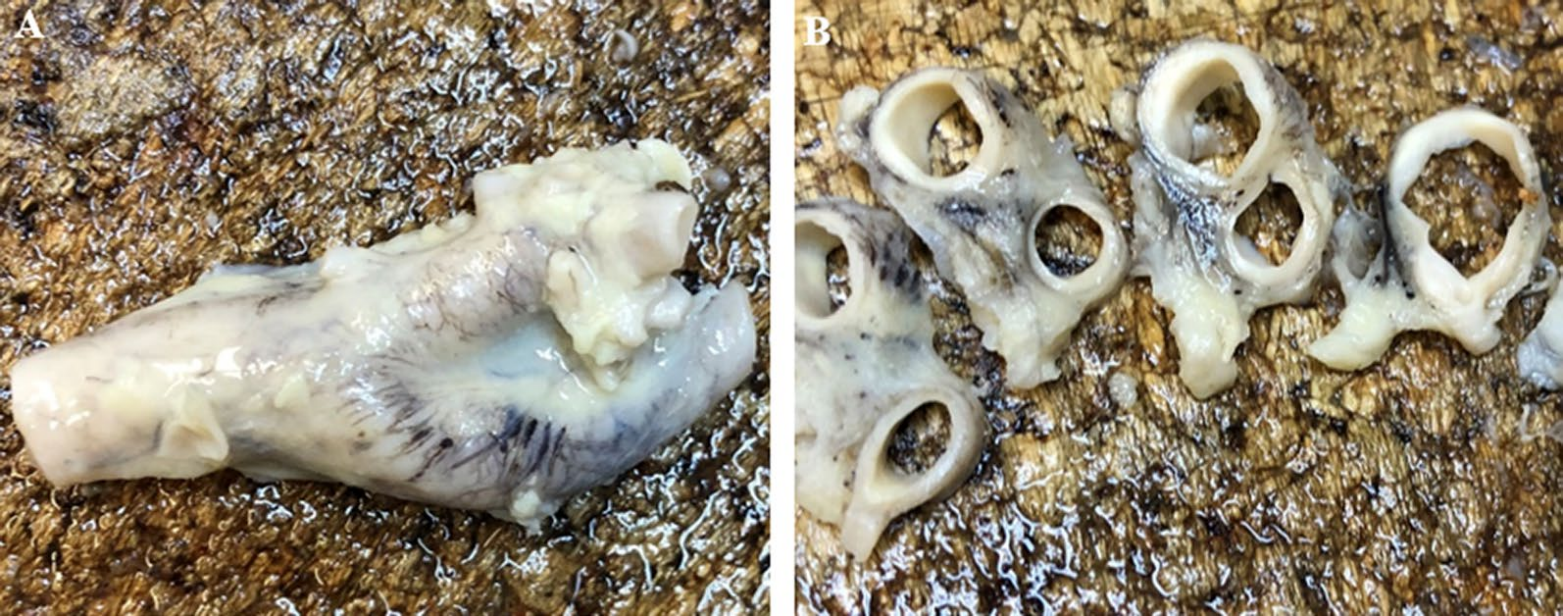
Carotid body within the bifurcation between the internal and external carotid arteries. (A) Haematoxylin–eosin (20 × magnification) and (B) Azan staining (20 × magnification). ACI: internal carotid artery; G: glomus caroticum.

**Figure 2.**
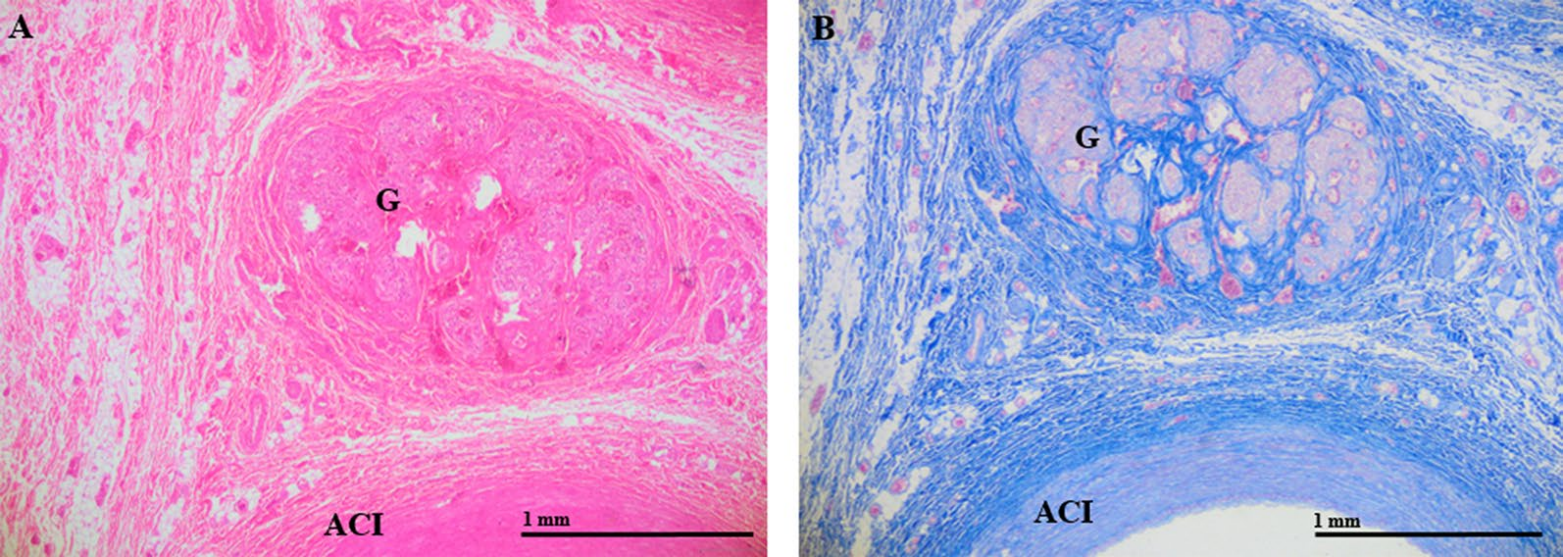
Carotid bifurcation after removal at autopsy and fixation in formalin (A) Excised carotid bifurcation. (B) Sections of the carotid bifurcation for embedding in paraffin wax.

The neck organs were removed in a bloodless condition (Figure 1). The left and right carotid bifurcations were collected at autopsy in each case. The common carotid was cut at 2 cm below the bifurcation, while the internal and external carotid arteries were cut at 2 cm above the carotid bifurcation. The excised carotid bifurcations surrounded by tissue were fixed in formalin (8%–10%). After fixation, the carotid bifurcations were laminated into thin slices (a few millimetres thick) cut in the horizontal plane (Figure 2) and then embedded in paraffin wax. Tissues were processed for haematoxylin–eosin staining, Azan and iron staining, and immunohistochemical detection of Hsp-27, Hsp-60, Hsp-70, and AQP-3. For immunohistochemical staining, sections were first pretreated with citrate buffer (pH 6.0) for 30 min at appropriate dilutions, according to the manufacturer’s instructions (Dako LSAB2 kit or Dako Envision + kit;). Sections were then incubated with mouse monoclonal antibodies against anti-Hsp 27 (ab2790; Abcam, Cambridge, UK), anti-Hsp60 (H4149; Sigma-Aldrich, Missouri, US), or anti-Hsp70 (ab2787; Abcam, Cambridge, UK) or a rabbit polyclonal antibody against AQP-3 (ab125219; Abcam, Cambridge, UK) overnight at 4 °C. Sections were then incubated in appropriate secondary antibodies for 60 min at room temperature (for Hsp antibodies) or 90 min at 37 °C (for the AQP-3 antibody).

All carotid bifurcations were examined by light microscopy (Leica DM 1000, Wetzlar, Germany) for the assessment of haemorrhage and expression of Hsp27, Hsp60, Hsp70, and AQP-3 in the tissues surrounding the bifurcations, the sinus, and the carotid body.

## Results

Among the 22 study group cases of strangulation causing death, there were 16 cases of hanging and 6 cases of ligature strangulation (Table 1). We hypothesised that the cases would have experienced differing degrees of neck force (e.g. because of the rope material, rope diameter, and hanging position [upright or seated]), which would cause different pathological findings. In all cases, the deaths were caused by accidental or suicidal strangulation. No evidence of homicide was observed.

**Table 1.** List of patients in the study group, including 22 cases with lethal ligature strangulation or hanging.

No.	Sex	Age (year)	Cause of death	Findings of the neck	Signs of venous stasis of the head	Weight of heart (g)	Stenosis bifurcation	Type of hanging
1	Female	21	Ligature strangulation with scarf in a seated position	Ligature mark of the skin	None	250	–	–
2	Male	53	Hanging with a cable at the door and a plastic bag over the head	Ligature mark of the skin, Fracture of the left superior horn of the thyroid cartilage with haemorrhage	None	320	–	Complete
3	Male	77	Hanging at a tree with a tension belt	Ligature mark of the skin	Petechiae	540	–	Incomplete
4	Male	10	Hanging with a scarf at the stair railing	Ligature mark of the skin	None	190	–	Incomplete
5	Female	34	Hanging with scarf and belt at the door frame	Ligature mark of the skin, fracture of the right superior horn of the thyroid cartilage with haemorrhage	Petechiae, cyanosis	390	–	Incomplete
6	Male	89	Hanging	Strangulation mark of the skin, haemorrhage in the anterior musculature of the neck, fracture of the left superior horn of the thyroid cartilage with haemorrhage	None	675	–	Complete
7	Male	50	Death in hospital after hanging with rope at balcony balustrade	Ligature mark of the skin, haemorrhage into both sternocleidomastoid muscles and deep layers of right musculature, haemorrhage around tissue of the left hyoid bone, fracture of the right superior horn of the thyroid cartilage with haemorrhage	Petechiae	370	–	Complete
8	Female	58	Hanging with nylon rope	Ligature mark of the skin	Petechiae	330	–	Incomplete
9	Female	91	Ligature strangulation with kitchen towel	Fracture of the right superior horn of the thyroid cartilage with haemorrhage	Petechiae	330	–	–
10	Female	59	Hanging with a belt at a cloth hook	Ligature mark of the skin, haemorrhage in the left sternocleidomastoid muscle	Petechiae	360	–	Incomplete
11	Male	54	Hanging with a rope at a supply pipe	Ligature mark of the skin, haemorrhage in the anterior musculature of the neck, fracture of the left superior horn of the thyroid cartilage	Petechiae	390	–	Complete
12	Male	48	Ligature strangulation with cable tie	Ligature mark of the skin	Petechiae, cyanosis	400	–	–
13	Male	45	Hanging with cable at the door frame	Cable around the neck, no ligature mark distinguishable because of putrefaction, fracture of the left superior horn of the thyroid cartilage	Not distinguishable because of putrefaction	300	–	Incomplete
14	Male	44	Hanging with nylon rope, fracture	Ligature mark of the skin, fracture of the right superior horn of the thyroid cartilage	Petechiae	420	–	Incomplete
15	Male	48	Ligature strangulation with rope	Ligature mark of the skin, haemorrhage in the left sternocleidomastoid muscle, fracture of the right superior horn of the thyroid cartilage	Petechiae, cyanosis	300	–	–
16	Male	58	Hanging at the window with a rope	fracture of the right superior horn of the thyroid cartilage with haemorrhage	Petechiae	250	–	Incomplete
17	Female	36	Hanging with rope	Ligature mark of the skin, haemorrhage subcutaneous tissue of the left posterior neck	None	400	–	Complete
18	Male	74	Ligature strangulation with fixation strap at the hospital	haemorrhage in the left anterior musculature of the neck	Petechiae	460	Stenosis of the carotid bifurcation	
19	Male	51	Hanging with rope at a ladder	Ligature mark of the skin, Haemorrhage at the insertion of the left sternocleidomastoid muscle at the clavicle	Petechiae, cyanosis	540	–	Incomplete
20	Female	88	Ligature strangulation with a chain barrier	Ligature mark of the skin, haemorrhage right stern mastoid muscle	None	260	Stenosis (20%) of the carotid bifurcation	–
21	Male	51	Hanging with a cable	Ligature mark of the skin, haemorrhage muscle of tongue right	Petechiae	390	–	Incomplete
22	Male	50	Hanging at the lattice bars of a window	Ligature mark of the skin	Petechiae	530	–	Incomplete

**Figure 3.**
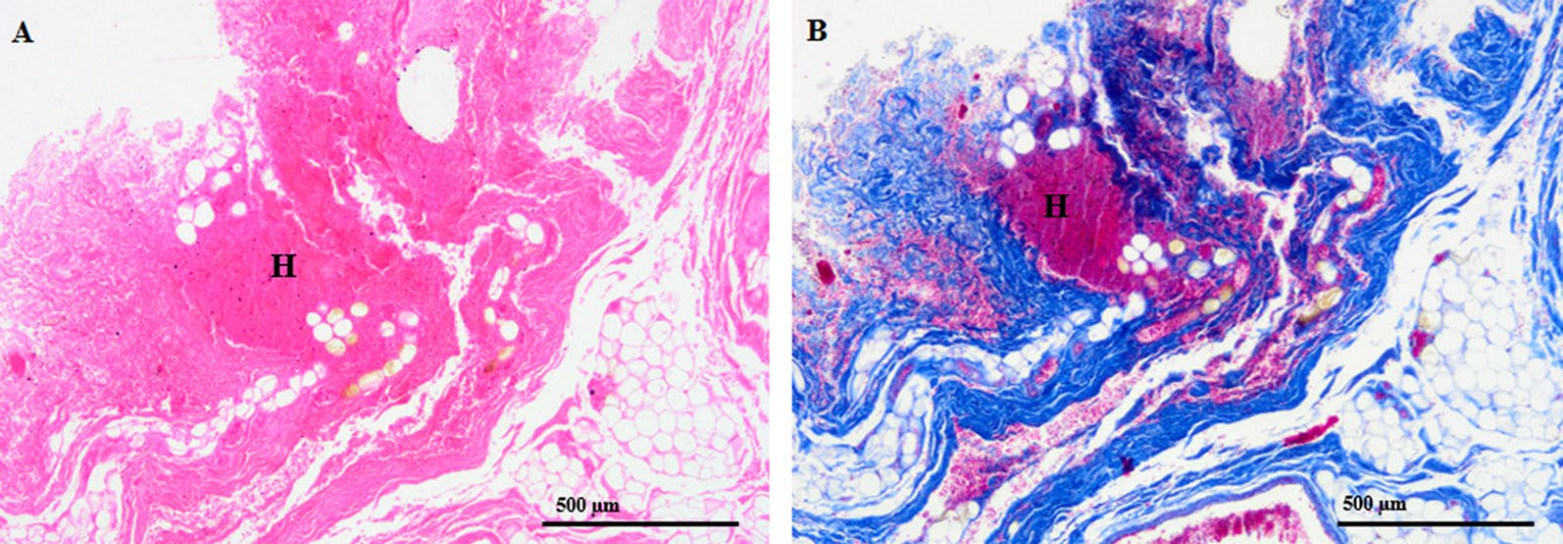
Representative case with mild haemorrhages (H) visualised with (A) haematoxylin–eosin (40 × magnification) and (B) Azan staining (40 × magnification) in the connective tissue, but not in direct proximity to the carotid bifurcation.

Of the 16 hanging cases, 3 had slight haemorrhage in the fat and connective tissues that were not in direct proximity to the carotid bifurcation (Figure 3). Fractures of the larynx and/or hyoid bone were observed in 8 cases, while signs of venous stasis of the head (e.g. petechiae) were observed in 11 cases.

**Figure 4.**
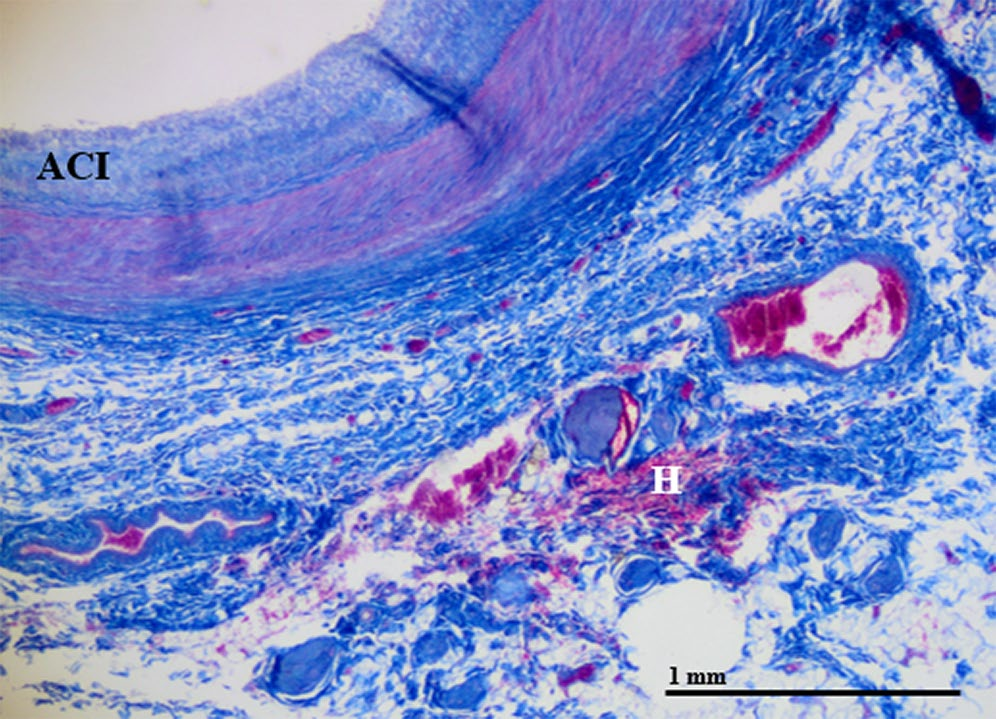
A small haemorrhage (H) near the arteria carotis interna (ACI) without relevant compression (20 × magnification).

Of the six cases with ligature strangulation, two cases showed a slight haemorrhage—haemorrhage occurred in the surrounding fat and connective tissues in one case and near the carotid bifurcation in one case (Figure 4).

Five cases showed small haemorrhages located predominantly in the surrounding fat and connective tissues. Therefore, we considered that the haemorrhages (including the haemorrhage in direct proximity to the carotid bifurcation) did not have any effects on the tissues such as severe pressure on the carotid body or sinus.

In the study group, there was no evidence of severe arteriosclerosis with vessel plaques. In four cases (cases 3, 6, 19, and 22), the heart exceeded the critical weight of 500 g, while cases 18 and 20 showed relevant carotid bifurcation stenosis. No cases showed any pathological alterations of the heart muscle. All iron stains were negative. The degree of iron-positive cells correlates with the age of the hematoma. A positive staining result that proves a vital haemorrhage requires survival for at least 3 days [18]. Thus, the negative iron staining indicated that the bleeding in all cases were rather fresh (acute bleeding) than bleeding that lasted few days.

**Figure 5.**
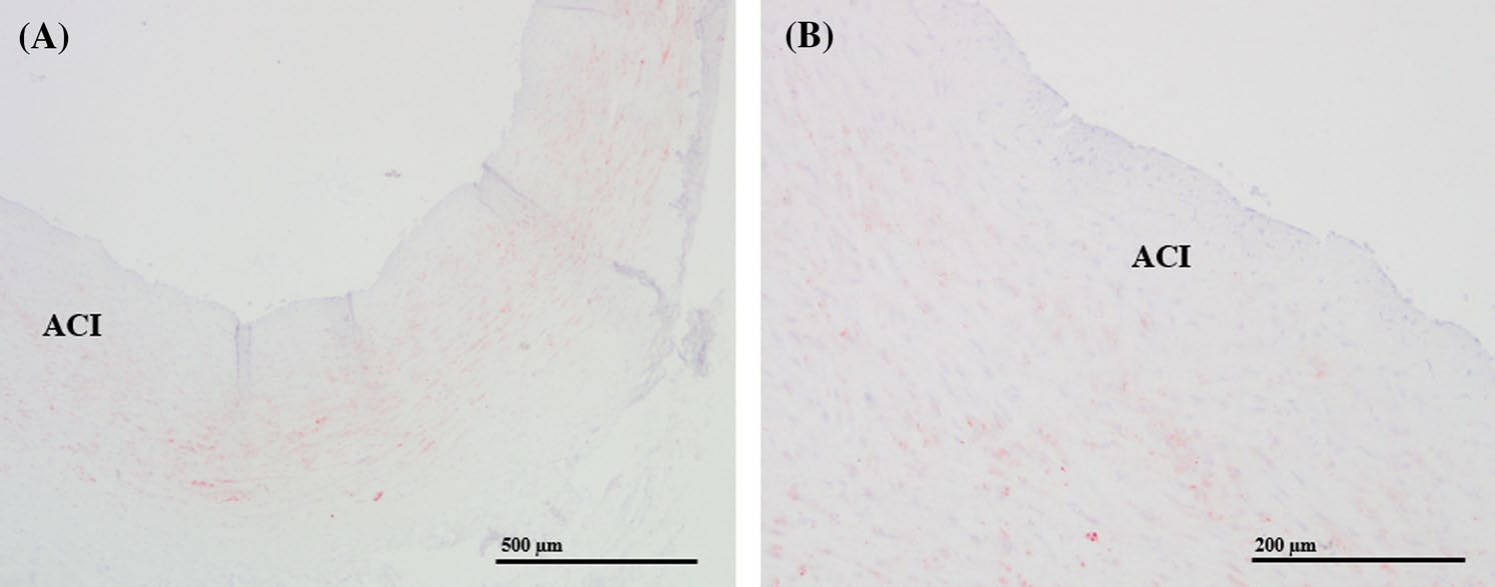
Five cases showed positive heat-shock protein-27 (Hsp-27) staining of the vessel wall of the internal carotid artery in 40 × magnification (A) and 100 × magnification (B).

A range of cellular stressors can increase Hsp expression [19]. For example, Hsps can be expressed within seconds or minutes during thermal stress,. Because of the temporal changes in expression of Hsps, they may be useful for determining the survival time after injury [20]. Because neck compression is also a form of external cellular stress to the neck tissues, including the carotid bifurcation, we assessed Hsp expression to examine the correlations with rapid death. However, only five cases showed positive Hsp-27 staining (Figure 5), all of which were cases of hangings. Three of these cases also showed pronounced signs of congestion, indicating a longer period before cardiac failure. No cases showed positive Hsp-60 or Hsp-70 staining. Overall, these findings suggest that neck compression has minimal effects on Hsp expression in carotid artery tissue.

**Figure 6.**
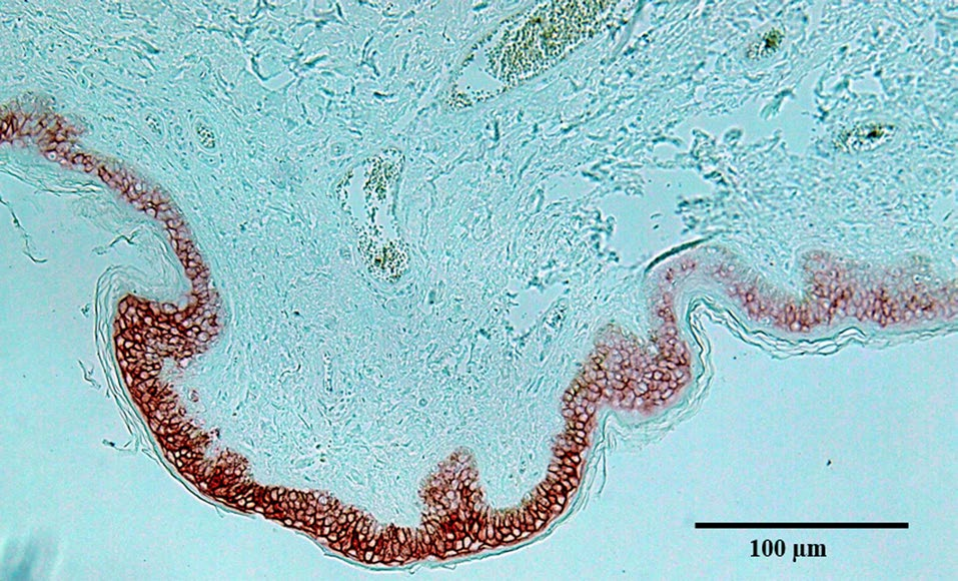
Positive aquaporin-3 staining in the epidermis of a ligature mark (200 × magnification). Source: [15].

AQP-3 is considered a valuable marker for antemortem neck compression in the skin [21] (Figure 6). Thereforewe assessed AQP-3 expression as a potential sign of relevant pressure on the tissue around the carotid bifurcation. However, no cases showed positive AQP-3 staining.

In the control group, no cases showed evidence of haemorrhage in carotid bifurcation tissues. Nevertheless, because of the age range (between 17 and 85 years) and variable pre-existing diseases, different stages of arteriosclerosis were found in this group.

## Discussion

Whether a death-causing carotid sinus reflex can exist has been of interest to forensic scientists and lawyers for decades because of its importance for criminal law [22]. In “The van Dielingen Case” from the 1930s, a man stated that he only briefly pulled his wife by her neckcloth, whereupon she immediately collapsed and died. Because there was no evidence of ligature strangulation, the man’s sentence was reduced to 2 years for bodily harm resulting in death [23].

Many forensic pathologists agree with the theory that stimulation of the carotid bifurcation can cause death. However, to date there is insufficient evidence to support this theory. There is wide acceptance that certain conditions must occur for cardiac reflex death following violence against the neck [24]. Other causes of death and pre-existing conditions including hypersensitivity caused by cardiac arrhythmia, digitalis medication, or atherosclerosis should be excluded [10]. Furthermore, a history of rapid death or immediate loss of consciousness should be considered, including signs of rapid cardiovascular system failure.

Two cases in our study group (74 and 88 years old) showed evidence of relevant carotid bifurcation stenosis. Sigrist et al. [25]were the first to report a diagnosis of sudden death after direct impact to the neck, with seven cases of rapid death due to reflex zone injury (Table 2). In their study, diagnosis required evidence of injury to the reflex zone at autopsy, as well as ruling out of signs of acute cardiovascular failure and other causes of death. By contrast, there was no evidence of rapid death in the history of our cases. In 20 of the 22 cases, autopsy revealed signs of venous stasis or muscular haemorrhage, indicating longer preservation of cardiocirculatory activity and vital reaction signs, which excludes immediate reflex death.

**Table 2.** Seven cases of rapid death after intensive impact and injury to the reflex zone of the neck.

Sex/age, occupation	Accident	Findings in the neck	Other findings	BAC (%)
Male/50 y, farmer	Hit by a hoof of a cow against the left side of the neck	Contusion of the skin, haemorrhage of the left neck, laceration of the intima of the carotid sinus, haemorrhage of the adventitia, and fracture of the upper horn of the left larynx	Acute cardiac dilatation, hyperaemia of all organs, and slight coronary arteriosclerosis FE: negative	Negative
Female/63 y, landlady	Fall to the ground, hit in the neck by the back of a chair	Haemorrhage in subcutaneous tissue and musculature in the front and side of the neck, intramural haemorrhage at the left carotid bifurcation, fracture of the left hyoid bone, and fracture of the right upper horn of the larynx	Acute cardiac dilatation, hyperaemia of inner organs, slight coronary arteriosclerosis, and arteriosclerotic shrinking of the right kidney FE: negative Toxicology: negative	Negative
Female/26 y, housewife	Fall from a motorbike, hit by the rim of a helmet against the neck	Abrasion at the chin and neck, haemorrhage of the neck soft tissue, and lacerations of both carotid arteries in the height of the bifurcation	Contusion of the lumbar soft tissue, rib fractures, lacerations of the lungs by rib fractures, and fractures of the arms FE: negative	Negative
Male/25 y, carpenter	Entrapment between the ground and edge of the roof of an overturned car	Abrasions of the neck, haemorrhage of the neck muscles, subadventitial haemorrhage of the left carotid artery, and subperiosteal haemorrhage at the larynx	Further abrasions at the head and hyperaemia of inner organs FE: negative	0.7
Female/20 y, unknown	Karate hit with the hand against the neck. Removal of the body	Fracture of the right upper horn of the larynx and slight haemorrhage	Criminal dismemberment of the body (no vital reactions) and putrefaction FE: not usable Toxicology: negative	0.3
Male/74, y farmer	Entrapment of the neck by a trailer of a tractor	Abrasion of the neck, subcutaneous contusions, slight haemorrhage of the neck muscle on the left side, and haemorrhage of the carotid sinus on both sides	Slight coronary arteriosclerosis and left ventricular hypotrophy FE: negative	0.54
Male/57 y, unemployed	Dispute in a home for men. Hit with a crutch against the neck and thorax	Haemorrhage of the neck in the front and on the left side, fracture of the larynx on the left side, laceration of the intima, and haemorrhage of the wall of the left carotid sinus	Lacerations on the head, chest, and extremities, advanced coronary arteriosclerosis, scar of myocardial infarction, bronchitis, pulmonary emphysema, and alcoholic liver damage FE: positive Toxicology: midazolam (25 ng/mL)	2.98

FE: pulmonary fat embolism; BAC: blood alcohol concentration. Source: [25].

The present study aimed to assess morphologically detectable injuries, particularly haemorrhages, in the region of the carotid bifurcation after ligature strangulation and hanging, which may have a relevant effect on the reflex zone. This type of haemorrhage was previously described in “The Deadly Broomstick Case”, in which a 51-year-old man was hit by a broomstick on the left side of his neck, after which he collapsed and died. Autopsy revealed a severe haemorrhagic compression of the carotid bifurcation. The authors suggested that this was a reflexogenic incident because of his immediate collapse. Because the haemorrhage surrounded the carotid bifurcation, and thus the carotid sinus, ongoing compression with a reflex causing prolonged cardiodepression were assumed. Without ongoing compression of the neck reflexogenic zones caused by the severe haemorrhage, his collapse following bradycardia and vasodilation may have been self-limiting [26].

Notably “The Deadly Broomstick Case” and each of the cases reported by Sigrist et al. showed quite intense violence against the neck (e.g. from a karate hit or trapping of the neck between a car and the ground), which is not comparable with violence against the neck resulting from hanging or ligature strangulation. Furthermore, all of our cases were suicidal or accidental strangulations, with no homicides, where a more severe impact of violence is expected. Additionally, the man from “The Deadly Broomstick Case” had pre-existing conditions including hypertension and arteriosclerosis, which may have caused his death (e.g. from arrhythmia) and which should be ruled out before a diagnosis of reflex death.

Neck haemorrhages can be tissue-displacing and produce ongoing stimulation of the carotid bifurcation, which can cause death. Nevertheless, unlike an enclosed cavity such as the skull, the surrounding neck tissue can expand following space-occupying events such as tumours or haematomas. Thus, extravasated haemorrhages in the neck area may not have sufficient capacity to affect the carotid sinus and cause reflex death. There were no haemorrhages detected in our cases. However, this is a relatively common finding in people who die from strangulation. Therefore, an absence of haemorrhage cannot exclude a neck compression or reflex death. The haemorrhages detected in the cases reported by Sigrist *et al.* and the “The Deadly Broomstick Case” are not unexpected because they experienced violent impact to the neck. A reflex death was assumed to have occurred in these cases because of the rapid onset of death, rather than the presence of haemorrhages.

In the present study, no cases showed any positive AQP-3 staining. AQP-3 is a major water channel associated with the skin and plays a major role in water reabsorption, including replacement of water loss due to evaporation. AQP-3 is expressed in the skin of ligature marks and is considered a reliable marker of antemortem neck compression [21]. AQP-3 is also expressed in other cell types including adipocytes, astrocytes, and endothelial cells [21]. Because AQP-3 can be induced in the skin following compression, we examined AQP-3 expression in the carotid bifurcation and surrounding tissue. However, our findings suggest that AQP-3 staining is not a useful marker for relevant neck pressure, or that there was actually no relevant neck impact. Further studies are required to confirm these findings, including more cases with different types of neck impacts and with signs of rapid death.

Finally, our findings provide no direct evidence for reflex cardiac death resulting from a brief force against the neck. Nevertheless, in selected cases with a suitable history, the potential for reflex death following intense force against the neck should still be considered. However, other causes of death should be ruled out by histological, morphological, and toxicological investigations, while morphological findings, including relevant haemorrhages in the carotid artery tissues and signs of prolonged survival and vital reactions, should be detected [25]. Notably an absence of haemorrhages does not rule out reflex death. Therefore, other markers are required to indicate a relevant pressure to the carotid bifurcation region. Without these conditions, a diagnosis of reflex death is not appropriate.

## Authors’ contributions

Julia Ulbricht carried out the histological analyses and drafted the manuscript; Elke Doberentz and Burkhard Madea conceived the study, participated in its design and coordination, and helped to draft the manuscript. All authors contributed to the final text and approved it.
